# Seasonal shifts in the gut microbiome indicate plastic responses to diet in wild geladas

**DOI:** 10.1186/s40168-020-00977-9

**Published:** 2021-01-23

**Authors:** Alice Baniel, Katherine R. Amato, Jacinta C. Beehner, Thore J. Bergman, Arianne Mercer, Rachel F. Perlman, Lauren Petrullo, Laurie Reitsema, Sierra Sams, Amy Lu, Noah Snyder-Mackler

**Affiliations:** 1 Department of Anthropology, Stony Brook University, Stony Brook, NY 11794 USA; 2 Department of Anthropology, Northwestern University, Evanston, IL 60208 USA; 3 Department of Psychology, University of Michigan, Ann Arbor, MI 48109 USA; 4 Department of Anthropology, University of Michigan, Ann Arbor, MI 48109 USA; 5 Department of Ecology and Evolutionary Biology, University of Michigan, Ann Arbor, MI 48109 USA; 6 Department of Psychology, University of Washington, Seattle, WA 98195 USA; 7 Interdepartmental Doctoral Program in Anthropological Sciences, Stony Brook University, Stony Brook, NY 11794 USA; 8 Department of Anthropology, University of Georgia, Athens, GA 30602 USA; 9 Center for Evolution and Medicine, Arizona State University, Tempe, AZ 85281 USA; 10 School of Life Sciences, Arizona State University, Tempe, AZ 85287 USA; 11 Department of Biology, University of Washington, Seattle, WA 98195 USA

**Keywords:** Gut microbiome, Graminivory, Seasonality, Thermoregulation, *Theropithecus gelada*, Primates

## Abstract

**Background:**

Adaptive shifts in gut microbiome composition are one route by which animals adapt to seasonal changes in food availability and diet. However, outside of dietary shifts, other potential environmental drivers of gut microbial composition have rarely been investigated, particularly in organisms living in their natural environments.

**Results:**

Here, we generated the largest wild nonhuman primate gut microbiome dataset to date to identify the environmental drivers of gut microbial diversity and function in 758 samples collected from wild Ethiopian geladas (*Theropithecus gelada*). Because geladas live in a cold, high-altitude environment and have a low-quality grass-based diet, they face extreme thermoregulatory and energetic constraints. We tested how proxies of food availability (rainfall) and thermoregulatory stress (temperature) predicted gut microbiome composition of geladas. The gelada gut microbiome composition covaried with rainfall and temperature in a pattern that suggests distinct responses to dietary and thermoregulatory challenges. Microbial changes were driven by differences in the main components of the diet across seasons: in rainier periods, the gut was dominated by cellulolytic/fermentative bacteria that specialized in digesting grass, while during dry periods the gut was dominated by bacteria that break down starches found in underground plant parts. Temperature had a comparatively smaller, but detectable, effect on the gut microbiome. During cold and dry periods, bacterial genes involved in energy, amino acid, and lipid metabolism increased, suggesting a stimulation of fermentation activity in the gut when thermoregulatory and nutritional stress co-occurred, and potentially helping geladas to maintain energy balance during challenging periods.

**Conclusion:**

Together, these results shed light on the extent to which gut microbiota plasticity provides dietary and metabolic flexibility to the host, and might be a key factor to thriving in changing environments. On a longer evolutionary timescale, such metabolic flexibility provided by the gut microbiome may have also allowed members of *Theropithecus* to adopt a specialized diet, and colonize new high-altitude grassland habitats in East Africa.

Video abstract

**Supplementary Information:**

The online version contains supplementary material available at 10.1186/s40168-020-00977-9.

## Background

Obtaining sufficient nutrients is a fundamental challenge for most animals. Yet, the availability and nutritional content of food can vary temporally and spatially in response to changes in climate and geography. Nutritional demands further vary in response to thermoregulatory needs and life history processes, such as growth and reproduction [1, 2]. Animals have evolved a variety of behavioral and physiological strategies to cope with these shifting demands, including altered feeding and activity patterns and increased mobilization of stored fat to fuel energetic demands [3–6]. Recently, the gut microbiome has been proposed as an additional avenue by which animals can cope with changing dietary landscapes and energetic challenges [7–9]. The gastrointestinal tract of animals harbors a dense microbial community that helps to break down and ferment plant structural carbohydrates, producing short-chain fatty acids (SCFAs) that can be used as an energy source by hosts [10–12]. The absorption of SCFAs in the gut may be particularly important for herbivorous species, such as foregut and hindgut fermenters, which obtain as much as 40-90% of their energy requirements from bacterial degradation of complex plant polysaccharides [13–16]. Additionally, variation in gut microbiome composition affects the efficiency of caloric harvest and the metabolic programming of the host [11, 17–20]. For instance, in mice (*Mus musculus*) and humans, obese and lean individuals have strikingly different gut microbiota composition, with obese phenotypes being associated with higher energy extraction from diet and increased lipogenesis [21–23].

In wild mammals, the gut microbiome responds rapidly to seasonal and dietary changes [7, 24–29], presumably to buffer seasonal energetic challenges [7, 26]. For example, a simultaneous increase in bacterial taxa involved in fiber fermentation and in SCFA concentrations during the dry season may allow Mexican black howler monkeys (*Alouatta pigra*) to maintain energy balance during energetic shortfalls without changes in activity or ranging patterns [7]. Moreover, gut bacteria increase intestinal absorptive capacity, energy homeostasis, and fat burning during cold periods in mice [30], and improve digestive efficiency and SCFA production in energetically challenged ruminants living in cold environments and at high-altitude [31, 32]. These microbial shifts likely come at some cost. For instance, increases in microbes that improve host metabolism under certain conditions may reduce the abundance of microbes that support host immune function [33, 34]. However, in seasonal and nutritionally challenging environments, enduring these trade-offs may be necessary for host survival and reproduction.

Geladas (*Theropithecus gelada*) represent an excellent system to investigate the relationship between gut microbiota composition and seasonal variation in host diet and energy needs. Despite being the only graminivorous primate with up to 90% of their diet comprised of grass [35, 36], their gastrointestinal tract appears poorly adapted to this specialization (but see [37, 38] for dental, manual, and locomotor adaptations), closely resembling their closest phylogenetic relatives, baboons (*Papio* spp.)—a taxon that is omnivorous [39]. To compensate, geladas may rely heavily on their gut microbiota to maximize nutrient extraction from grasses, likely through hindgut fermentation [39, 40]. Moreover, geladas live in a high-altitude, energetically demanding environment that exhibits marked inter- and intra-annual fluctuation in rainfall and temperature [35, 41]. During rainier months, when grass is abundant, they forage almost exclusively on aboveground graminoid leaves and seeds, and during drier months, when grass availability decreases, they shift heavily to underground foods (rhizomes, roots, corms, bulbs) [35, 42]. This diet provides distinct challenges. Underground foods are considered a fallback food for geladas since they take additional time and effort to harvest, are harder to process, and are relied upon only when grasses are less abundant [35, 38]. Despite being considered a fallback food, these underground foods are rich in starches and carbohydrates, suggesting that they contain more nutritional energy than grass [43]. This high amount of energy, however, comes at some cost: roots and rhizomes are generally higher in fibers and lignin—and thus harder to digest than grasses. In addition to these nutritional challenges, ambient temperatures frequently drop to near freezing, and the metabolic costs of thermoregulation are known to strongly influence gelada physiology [44] and the timing of reproduction [41, 45]. Thus, seasonal dietary shifts and temperature variation may lead to distinct digestive and thermoregulatory challenges.

One previous study on geladas from Guassa, Ethiopia, found that gut microbial communities of adult females shifted across seasons [40], supporting the hypothesis that the gut microbiome may help hosts confront environmental challenges. This study focused on adult females and assessed seasonal variation by separating the samples into two categorical seasons (i.e., rainy, dry). Our study expands on this study by including adult males, incorporating continuous climatic data across several years, and examining proxies of thermoregulatory stress (in addition to diet) as factors that can influence the composition and function of the gelada gut microbiome. Indeed, rainfall and temperature vary independently of each other and represent distinct ecological challenges in gelada ecosystems. Therefore, we were interested in further testing which aspect of gelada ecology more strongly determines seasonal microbiome shifts.

We analyzed the gut microbiome composition and predicted microbiome function in 758 fecal samples across 5 years from 48 adult male and 86 adult female geladas living in the Ethiopian highlands in the Simien Mountains National Park. The Simien Mountains Gelada Research Project (SMGRP) has been collecting detailed climatologic, demographic, and behavioral data from this study population since 2006, allowing us to examine how ecological (rainfall and temperature) and individual (group membership, sex, reproductive status, and age) factors influence gelada gut microbiome composition. We hypothesized that ecological factors would be more strongly associated with variation in the gelada microbiome than individual factors, and that rainfall and temperature would have independent effects. In particular, we expected that rainfall, which is a good proxy for grass availability [35], would have the strongest effect on the gelada gut microbiome. We predicted that the taxonomic changes associated with rainfall would mainly reflect a shift to grass-based versus underground food-based diet, in order to allow individuals to maximize energy extraction from those seasonal foods. We found that the gelada microbiome exhibited drastic shifts related to climatological variables; but individual variables, like age and sex, had minimal effects. Rainfall and temperature exerted independent effects on the microbial composition and predicted function—with rainfall having a stronger effect on the gelada gut microbiome. High rainfall, which is correlated with grass availability [35], was associated with more cellulolytic and fibrolytic bacterial taxa, when graminoid leaves were the main food source. Dry periods, when geladas consume more underground foods [35], were associated with more amylolytic and methanogenic taxa. Cold periods were further characterized by more amylolytic taxa, and hot periods by more methanogenic taxa. In both drier and colder periods, the gut microbiome shifted to predicted functions that suggested increased digestive efficiency, including energy, amino acid, and lipid metabolism. Overall, gelada gut microbial composition covaried with diet and temperature in a pattern that suggests plastic but distinct responses to dietary and metabolic challenges.

## Results

### The gelada gut microbiome

**Fig. 1:**
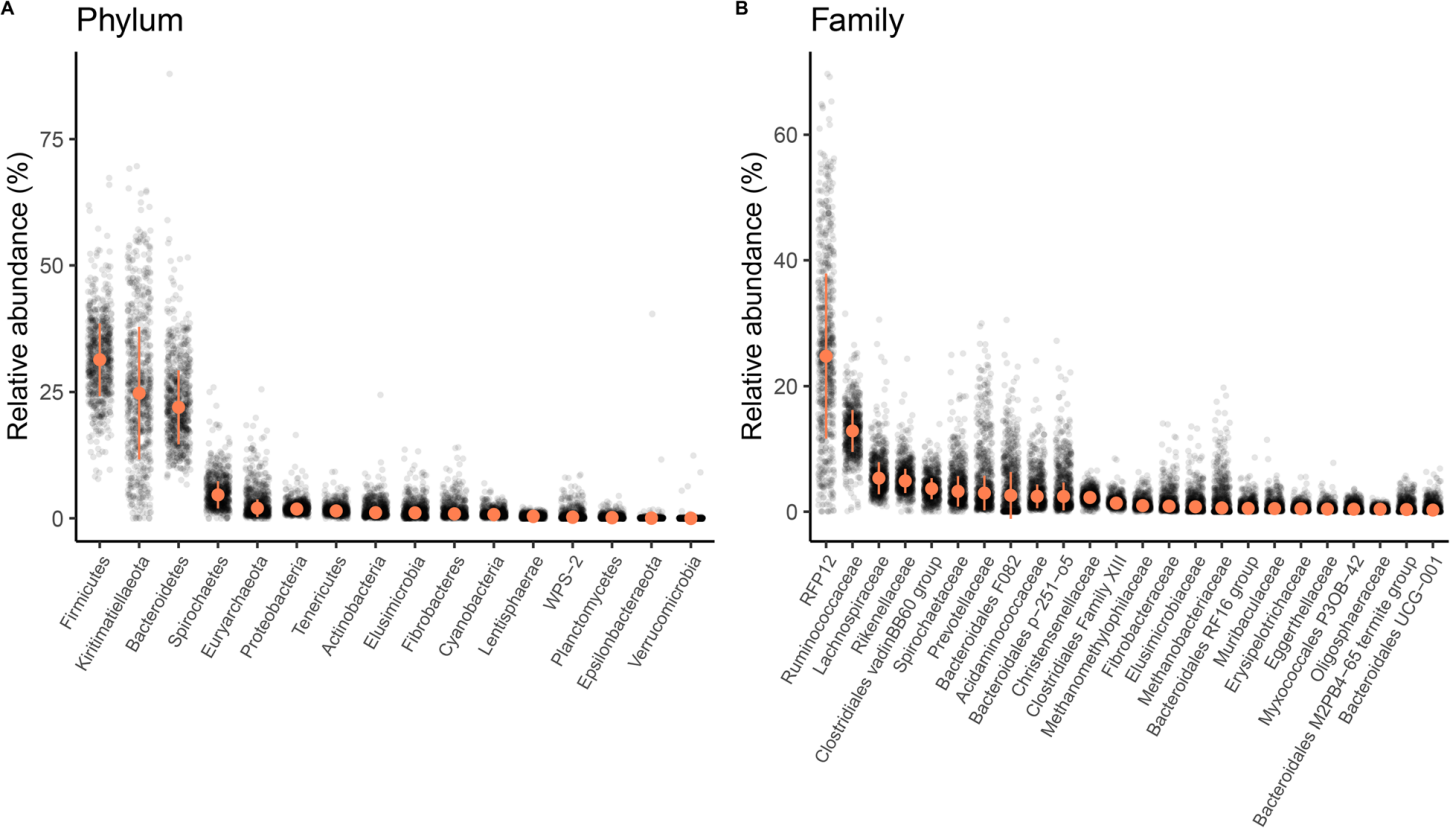
Taxonomic composition of the gelada gut at the phylum and family levels. Relative abundance (**a**) of all bacterial phyla and (**b**) of the 24 most abundant families (relative abundance> 0.02%) in the gelada feces. The median and median absolute deviation (error limit) are represented in orange.

We identified 3295 amplicon sequence variants (ASVs) in 758 fecal samples (mean ± SD = 813 ± 243 ASVs per sample, range = 92-1730) using deep 16S rRNA gene amplicon sequencing. Most ASVs could be taxonomically assigned to the phylum (100%), class (99%), and order level (99%), but assignments decreased substantially at the family (85%) and genus (61%) levels. These 3295 ASVs came from 16 different phyla, 65 families, and 200 genera (Table S1, Fig. 1, Figures S1-S2). Of the 3295 ASVs, 170 (5%) were present in at least 90% of samples and form what can be considered the “core microbiota” of geladas (Table S2). The four most abundant bacterial phyla were *Firmicutes* (32%), *Kiritimatiellaeota* (formerly called *Verrucomicrobiota subdivision 5*; 26%), *Bacteroidetes* (23%), and *Spirochaetes* (5%) (Table S1, Fig. 1a). All microbes assigned to *Kiritimatiellaeota* were part of the *RFP12* family and represent almost one quarter of the gelada gut microbiome (mean 26%, range 0.02-70%, Fig. 1b). Despite the abundance of *RFP12*, the 78 ASVs belonging to this group could not be assigned below the family level. We therefore queried these sequences against the NCBI database using BLAST [46, 47]. These *RFP12* sequences were most similar (~ 97%) to bacterial sequences previously found in the feces of hindgut herbivorous species (Asiatic elephant, domesticated horse, Somali wild ass, zebra, and black rhinoceros) [48] and, to a lesser extent, the rumen of several foregut fermenters (cattle, yak, red kangaroo; Table S3). Taken together, this indicates that the *RFP12* family is a key microbe for herbivorous species, particularly hindgut fermenters, and likely plays an important fermentative role in plant digestion in geladas. Other taxa found at high frequency in the guts of ruminants and herbivorous hindgut fermenters were also prevalent in the gelada gut, including many cellulolytic/fibrolytic (13% *Ruminococcaceae*, 6% *Lachnospiraceae*, 4% *Clostridiales vadinBB60 group*, 1.5% *Fibrobacteraceae*) and fermentative families (5.3% *Rikenellaceae*, 5% *Prevotellaceae*, 4.1% *Bacteroidales F082*) (Table S1, Fig. 1b and S1). The *Spirochaetes* phylum was mostly composed of *Treponema* (3.5%), a genus involved in lignocellulose degradation [49].

### Dietary changes

**Fig. 2:**
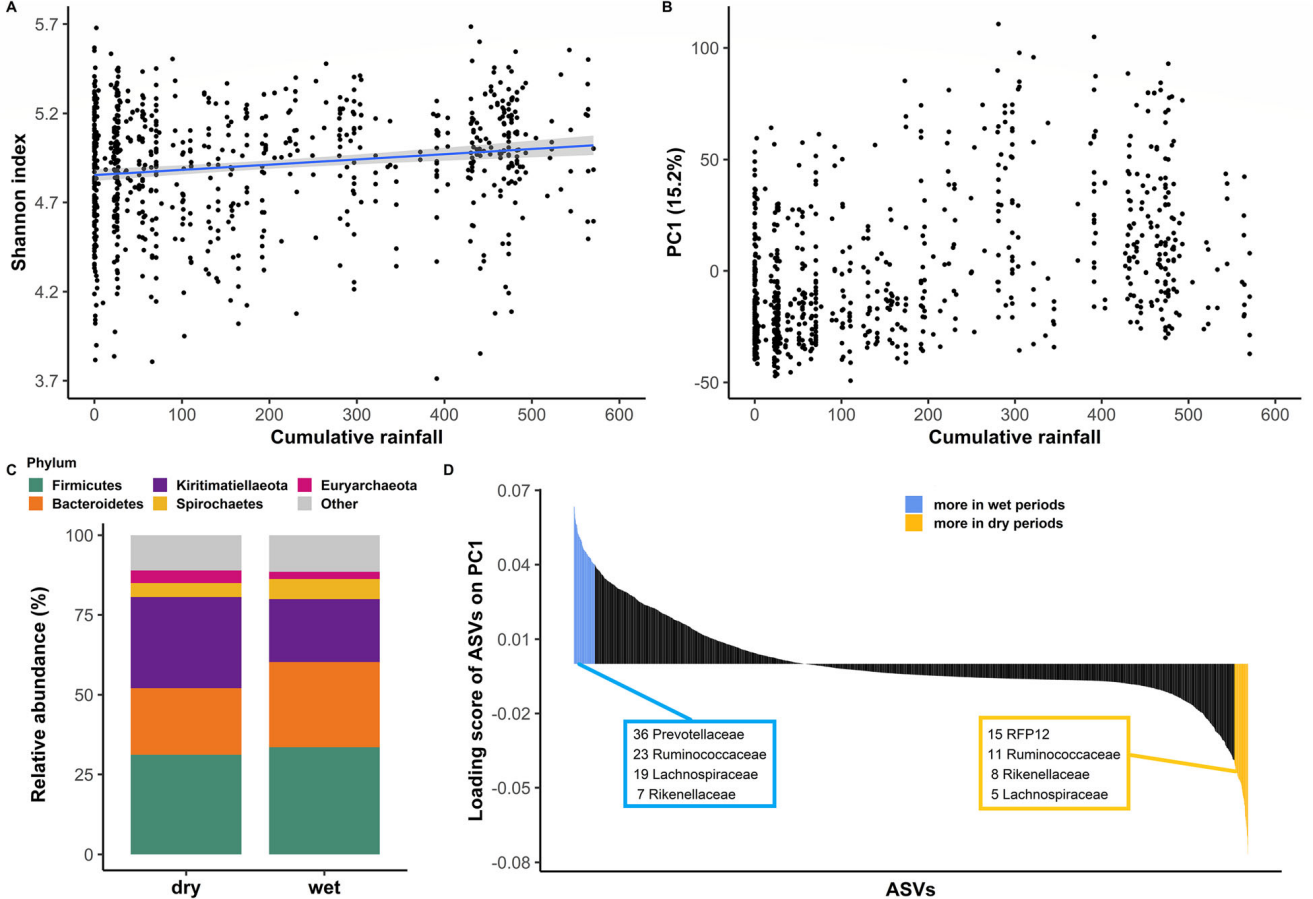
Rainfall structures the gelada gut microbiome. **a** Partial residual plot of Shannon alpha diversity index according to cumulative rainfall (in mm). Black dots represent the partial residuals from the LMM (i.e., showing the association between cumulative rainfall and alpha diversity, while controlling for all other predictors). The blue line and confidence intervals come from a linear regression (for representation only). Seven outlier samples (with a particularly low Shannon index) were omitted for clarity of representation. **b** Visualization of between-sample dissimilarity (based on Aitchison distance) on the first principal component (PC1) according to cumulative rainfall. **c** Compositional barplot of the five most abundant phyla in the dry (< 100 mm of rain in the past month, *N* = 362) and wet (> 200 mm of rain in the past month, *N* = 282) samples (cumulative rainfall was converted to a categorical variable for representation purposes). **d** Loading scores of each amplicon sequence variant (ASV) on the first principal component. ASVs with a loading score > 0.4 (characteristics of the wet season) and < −0.4 (characteristic of the dry season) are colored.

To examine how seasonal variation in rainfall and temperature was associated with changes in the gelada gut microbiome, we used measures of true climatic conditions, including monthly cumulative rainfall (an appropriate proxy of grass availability in the Simiens: [35]) and average monthly minimum temperature (a proxy of thermoregulatory constraint: [41, 44]). At the level of within-sample community diversity (“alpha diversity”), we found that cumulative rainfall was positively associated with Shannon evenness (Table 1, Fig. 2a, c) but had no effect on bacterial richness or Faith’s phylogenetic diversity (Table S4, Figure S3). Thus, rainfall was associated with the relative abundance of ASVs within a sample but not the absolute number of ASVs or their phylogenetic diversity.

**Table 1:** Determinants of alpha diversity, as measured by the Shannon index

Fixed factor	Estimate	SE	95% confidence interval	LRT	*p* value
**Sex (male)**	−0.12	0.04	[−0.20; −0.05]	9.27	**0.002**
Age	0.01	0.02	[−0.02; 0.04]	0.49	0.484
**Cumulative rainfall**	0.05	0.02	[0.02; 0.08]	12.13	**< 0.001**
Minimum temperature	0.00	0.01	[−0.03; 0.03]	0.04	0.848
**Sequencing depth**	0.07	0.01	[0.04; 0.10]	24.24	**< 0.001**

Parameters and tests are based on linear mixed models of 758 samples and 131 individuals, controlling for individual identity and unit membership. Factors with *p* values less than 0.05 are highlighted in bold

Cumulative rainfall significantly explained 3.3% of the overall compositional dissimilarity—or beta diversity—between samples (as measured by Aitchison distance) (Table 2), which was less than that explained by two demographic variables: individual identity and unit (social group) membership (20% and 6%, respectively; Table 2). The first principal component of beta diversity, which explained 15% of variation, was strongly associated with rainfall (*r* = 0.43, *t* = 12.93, df = 756, *p* < 0.001, Fig. 2b). The ASVs that loaded positively on PC1 (i.e., correlated with higher rainfall, Fig. 2d) were primarily from the families *Prevotellaceae*, *Ruminococcaceae*, and *Lachnospiraceae* (Table S5 and S6). By contrast, the ASVs that loaded negatively on PC1 (i.e., more abundant in low rainfall, Fig. 2d) belonged to the family *RFP12* and a different subset of *Ruminococcaceae* that were not abundant during the wet season (Table S5 and S6).

**Table 2:** Determinants of beta diversity. Results of PERMANOVA testing for the predictors that significantly structure the gut microbiome of geladas, using 10,000 permutations and the Aitchison dissimilarity distance between samples. The *R*-squared values indicate the amount of between-sample variation explained by each variable.

Factor	*R*^2^ (%)	*p* value
**Individual**^a^	20.25	**< 0.001**
**Sequencing depth**^b^	3.77	**< 0.001**
**Unit**^b^	5.84	**< 0.001**
**Cumulative rainfall**^b^	3.30	**< 0.001**
**Minimum temperature**^b^	0.33	**< 0.001**
**Sex**^b^	0.23	**0.012**
Age^b^	0.19	0.045

^a^We first fit a model with individual identity as the only predictor in a PERMANOVA to estimate the sole effect of individual identity at explaining the overall gut composition of samples

^b^We then fit a second PERMANOVA model where all other predictors were fit, stratifying on individual identity to control for pseudoreplication of samples from the same individual

**Fig. 3:**
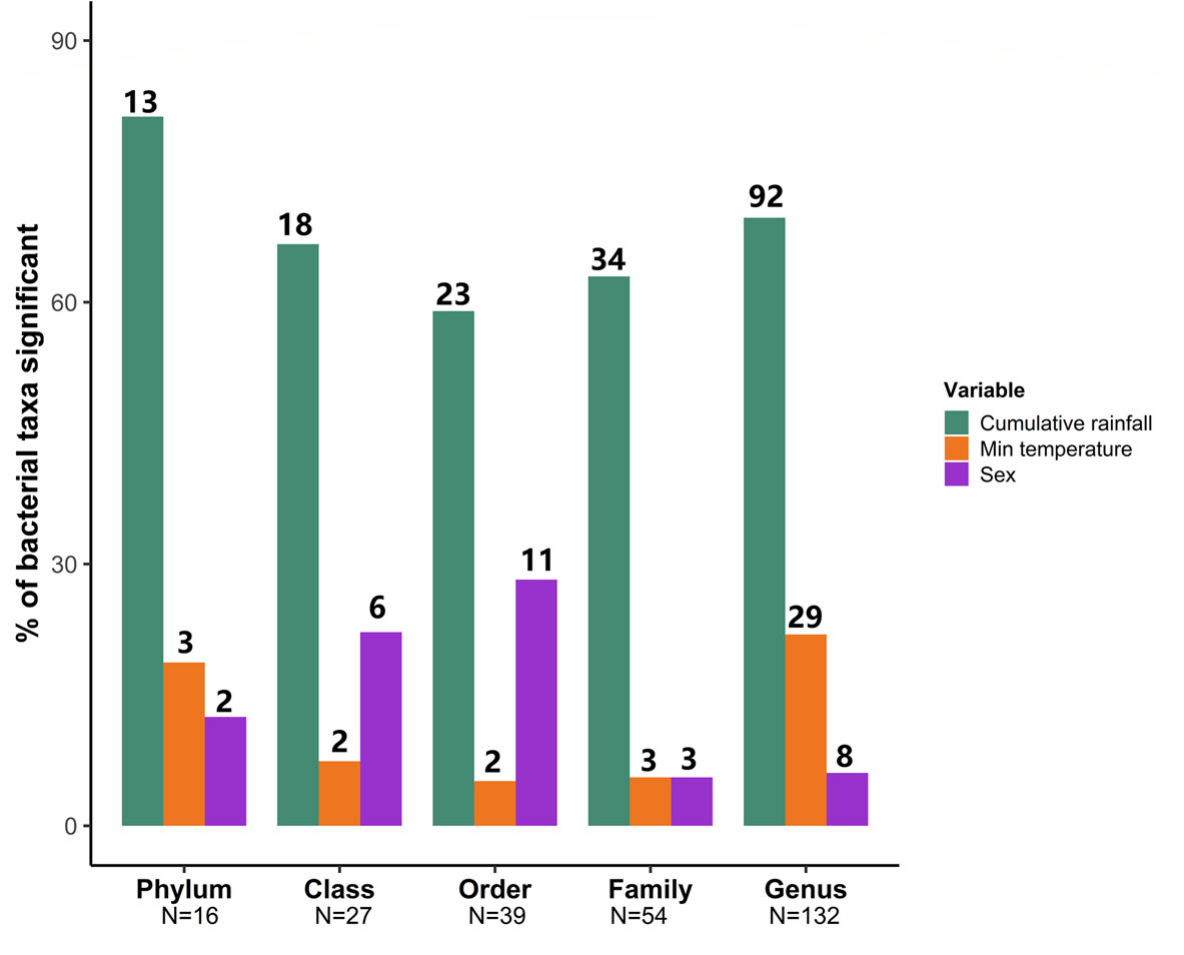
Rainfall exerts the strongest effect on bacterial relative abundance. Percent of taxa that are significantly associated (Benjamini-Hochberg corrected *p* values: *p*_BH_ < 0.05) with rainfall (purple bars), temperature (orange bars), or sex (green bars), across five taxonomic levels. For a given bacterial taxon, the significance of each predictor was assessed using a negative binomial GLMM of the count of this taxon per sample (controlling for sequencing depth as an offset factor, and including individual and unit membership as random effects). Only taxa with *p*_BH_ < 0.05 were considered significant. The numbers above the bars depict the number of taxa significantly differentially abundant, while the numbers below indicate the total taxa measured per level. Age was not significantly associated with relative abundance of any taxa at any level

**Fig. 4:**
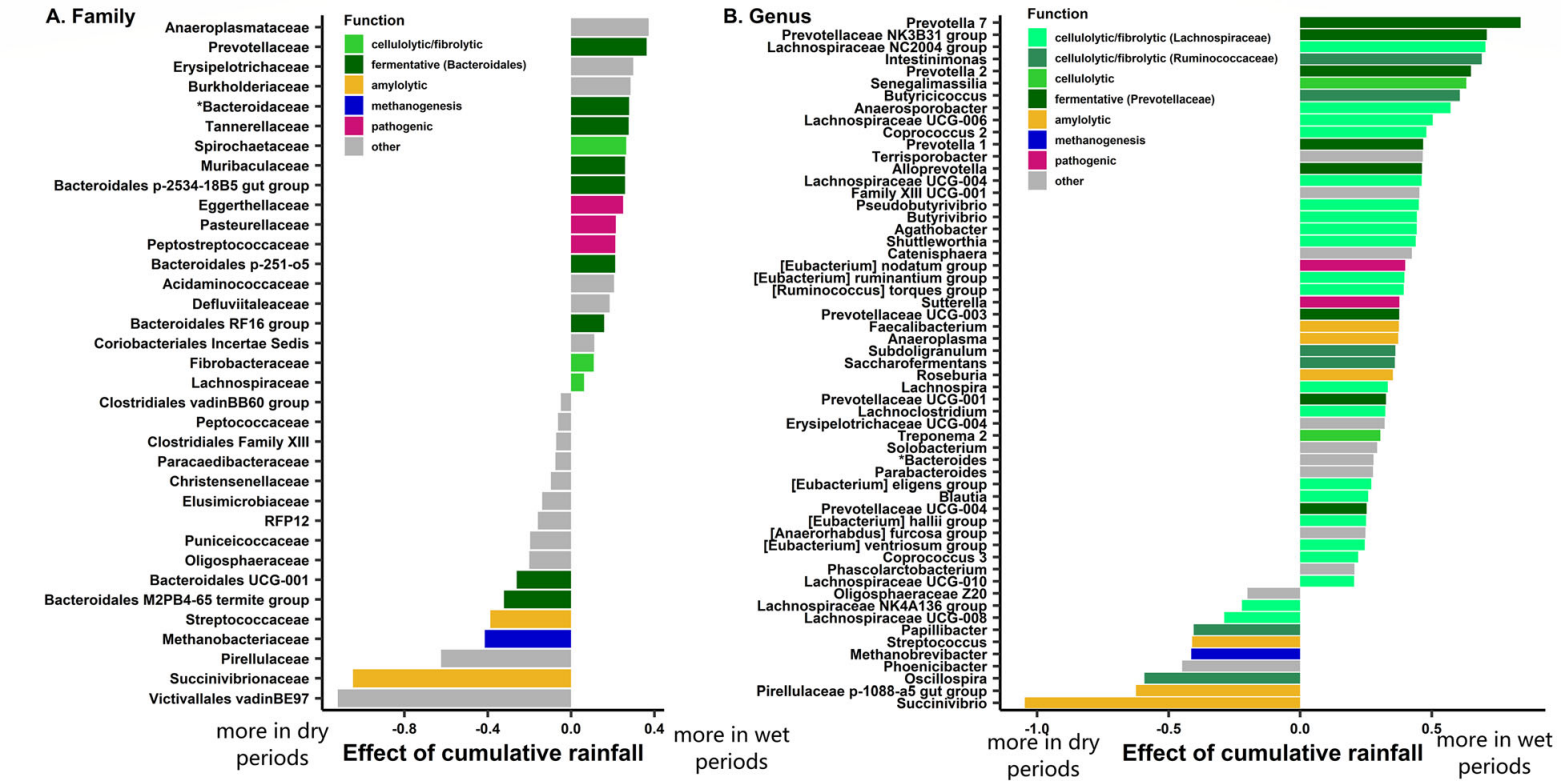
Rainfall predicts the relative abundance of many bacterial taxa. **a** Families and **b** Genera that are found differentially abundant according to cumulative rainfall. The estimate of the cumulative rainfall effect for each taxon comes from a negative binomial GLMM modeled separately for the counts of each taxon across  samples (controlling for sequencing depth as an offset factor, and including individual and unit membership as random effects). Taxa starting with “*” were fit with a binomial model instead. Only taxa with *p*_BH_ < 0.05 were considered significant. For ease of representation on panel B, only genera with effect sizes > |0.2| are represented. The full list of differentially abundant genera can be found in Table S7. Assignment of the “broad function” of a family or genus is for representation only, and is a simplification of the various functions subsumed within each taxonomic group.

**Fig. 5:**
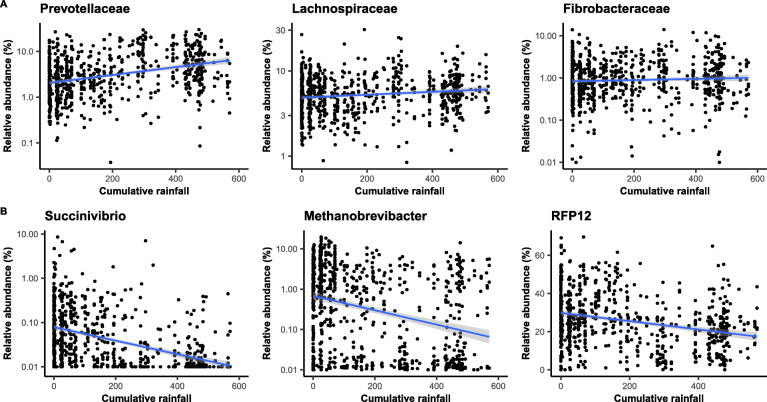
Relative abundance in six bacterial taxa (family or genus) that are significantly associated with rainfall. **a** Families more abundant during the wet season and **b** Families more abundant during the dry season. Note that the tick marks on the *y*-axis are spaced on a log10 scale (except for RFP12 which is plotted on a raw scale because of its high abundance). The blue line and confidence intervals come from a linear regression (for representation only). The significance of those effects has been estimated using negative binomial GLMMs including individual and unit membership as random effects

Cumulative rainfall predicted the relative abundance of gut microbes at all taxonomic levels and was significantly associated with the relative abundance of 63% of bacterial families tested (59-81% of taxa at other taxonomic levels, Fig. 3, Benjamini-Hochberg corrected *p* values: *p*_BH_ < 0.05). Thus, across most taxa, there was a clear contrast in the relative abundance of gut bacteria between the wet and dry periods (Table S7, Fig. 4). In wetter periods, there was an increase in several important fermentative families from the *Bacteroides* order (including *Prevotellaceae* and *Bacteroidaceae*), as well as in several cellulolytic/fibrolytic taxa (*Lachnospiraceae*, *Fibrobacteraceae*, *Spirochaetaceae*, and several genera from the *Ruminococcaceae*; Figs. 4 and 5a), suggesting improved digestive efficiency of plant cell wall polysaccharides at a time when the gelada diet consists mainly of grasses. In particular, nine *Prevotella* genera as well as the *Bacteroides* genus were at higher abundance during wetter periods than drier periods (Table S7, Fig. 4b). There was also an increase in several proficient cellulolytic genera (e.g., *Senegalimassilia*, *Butyrivibrio*, *Saccharofermentans*, *Cellulosilyticum*, *Marvinbryantia*) (Table S7, Fig. 4b). By contrast, the dry season was characterized by an increase in amylolytic genera (*Succinivibrio*; *Streptococcus* and *Pirellulaceae p-1088-a5 gut group*), in several efficient sugar-fermenting families (*Victivallales vadinBE97*, *Christensenellaceae*), and in the methane-producer *Methanobrevibacter*, a genus known to increase the rate of fermentation and digestive efficiency (Table S7, and Figs. 4 and 5b). Consistent with our beta diversity analyses, we also found an increase in the relative abundance of the *RFP12* family during the dry season (Table S7, Fig. 5b).

**Fig. 6:**
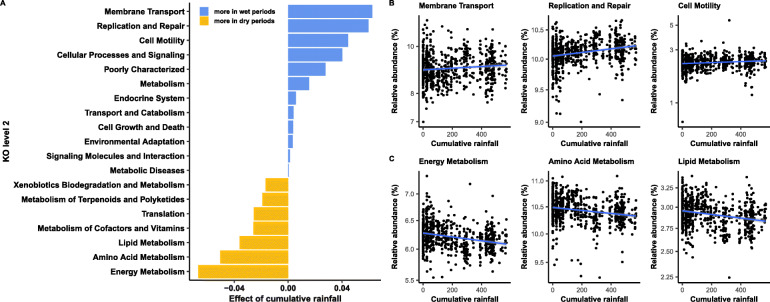
Rainfall predicts the functional profile of the gut microbiome. **a** Bacterial pathways at level 2 of KEGG Orthology (KO) that are differentially abundant according to cumulative rainfall (in mm). The estimate of the “rainfall” effect for each pathway comes from a LMM fitted on the relative abundance of each pathway per sample. Only pathways with *p*_BH_ < 0.05 are reported. Relative abundance of the three most enhanced functional pathways during **b** the wet season and **c** the dry season according to monthly cumulative rainfall. Note that the tick marks on the *y*-axis are spaced on a log10 scale. The blue line and confidence intervals come from a linear regression (for representation only). The significance of the rainfall effect effects per pathway was estimated using LMMs including individual and unit membership as random effects

The taxonomic changes associated with rainfall also corresponded to changes in the predicted function of the gelada gut microbiome (as assessed by PICRUSt2: [50]). During wetter periods, functional changes tended to reflect the activity of the cellulolytic and fermentative bacterial taxa. Microbial pathways involved in the transport of molecules through bacterial membranes (e.g., ions, sugars, lipids, peptides), DNA replication and repair, and cell motility (Tables S8-S9, Figs. 6, S4 and S5A) increased. We also found an increase in the metabolism of sugars (e.g., starch and sucrose metabolism, fructose, mannose, and galactose) (Figure S4 and S5A). Such activity probably reflects the exportation of sugar-cleaving enzymes and cellulosome complex across the outer membrane [12, 51] of fibrolytic bacteria (complex polysaccharides are too big to penetrate directly inside bacteria and have to be cleaved first) and the absorption of the soluble oligosaccharides back across the bacterial membrane [12, 51].

During drier periods, the gelada gut harbored a greater abundance of bacterial genes involved in energy, amino acid, and lipid metabolism (Tables S8-S9 and Fig. 6a, c). In particular, cellular energy production and cellular activity were enhanced during this period, as evidenced by increases in pathways involved in the citric acid cycle, oxidative phosphorylation, and fatty acid synthesis and metabolism (Figure S4 and S5B). Other energy metabolism pathways also increased during drier periods, including the methane pathway and the carbon fixation pathways, which are important for generating energy in anaerobic bacteria (Figure S4 and S5B). Finally, drier periods were associated with an increase in functions related to the synthesis of proteinogenic amino acids (e.g., tryptophan), the translation and synthesis of proteins (Figure S4), and the synthesis of lipopolysaccharide.

Note, however, that the average weighted Nearest Sequence Taxon Index (NSTI) value across all samples (a measure of how similar bacteria from a sample are to reference genome sequences) was relatively high in our sample (mean ± SD = 0.60 ± 0.13) compared to other mammals [50]. Our results should therefore be interpreted with caution, in that they provide a general idea of the predicted gene family profiles based on bacteria that were functional characterized in previous studies.

### Temperature

Compared to rainfall, minimum temperature had a much smaller impact on the gut microbiome. Average minimum temperature did not influence any metric of alpha diversity (Table 1 and S4, Figure S6A), and explained only 0.33% of the variation in beta diversity (Table 2, Figure S6B). Changes in temperature were significantly associated with the relative abundance of 5% of the families (5-22% at other taxonomic levels; Fig. 3; *p*_BH_ < 0.05). More specifically, colder temperatures were characterized by a greater abundance of two amylolytic genera (*Lactobacillus* and *Streptococcus*); in several sugar-fermenting (*Hydrogenoanaerobacterium*, *Clostridium sensu stricto 1*, *Coprococcus 1*) and cellulose-degrading bacteria (*Marvinbryantia* and two genera from the *Ruminococcaceae* family) (Table S7, Figure S7). By contrast, hotter temperatures were associated with an increase in *Verrucomicrobia*, in the methane-producer *Methanobrevibacter*, and in several cellulolytic/fibrolytic genera from the *Ruminococcaceae and Lachnospiraceae* families (Table S7, Figure S7).

Similar to our taxonomic analysis, we found that temperature had a much smaller effect on the predicted function of the gelada gut microbiome (Tables S8-S9, Figure S8). During colder periods, we found a predicted increase in bacterial pathways involved in lipid metabolism and energy production (notably in oxidative phosphorylation pathway; Figure S8). Other pathways that increased during colder periods involved DNA repair and recombination and the bacterial secretion system. During hotter weather, pathways were more poorly characterized and less specific, with predicted increases in methane metabolism and ABC transport (a membrane transporter).

### Sex, reproductive state, and age

The gut microbiome of females exhibited higher alpha diversity compared to males, regardless of the metric (richness, evenness, and Shannon index) (Table 1 and S4, Figure S9A). Across samples, however, sex explained little between-sample variation (i.e., < 1%) (Table 2, Figure S9B). We detected only a handful of bacterial taxa that were differentially abundant according to sex (Table S7, Fig. 3). At the phylum level, females harbored more *Verrucomicrobia* and *Proteobacteria* (particularly from class *Gammaproteobacteria*, *Deltaproteobacteria*, and *Alphaproteobacteria*). At the family and genus levels, females had more taxa involved in lactic acid metabolism (*Lactobacillaceae*, *Anaerovibrio*), cellulolysis (*Saccharofermentans*), and regulation of glucose and fat transport (*Erysipelatoclostridium*). Males, on the other hand, only harbored more *Pirellulales*. No predicted metabolic pathway differed in abundance between males and females (Tables S8-S9).

Female reproductive state did not influence any alpha diversity metric (Table S10, Figure S10A) and was not a significant factor influencing beta diversity between samples (Table S11, Figure S10B). Very few taxa were differentially abundant according to female reproductive state (Table S12 and S13). Pregnant females harbored more *Verrucomicrobiota* (class *Verrucomicrobiae*) and *Epsilonbacteraeota* than cycling and lactating females (Table S13). In particular, the genus *Helicobacter* (within the family *Epsiolonbacteroaeto*)—a presumed pathogen—was highly prevalent in pregnant females (Table S13). No predicted metabolic pathways were found to differ based on reproductive state (Table S14-S15). Age did not influence any metric of alpha diversity (Table 1 and S4, Figure S11A) or beta diversity (Table 2, Figure S11B), and no bacterial taxa (Table S7) or predicted metabolic pathways (Table S8-S9) were differentially abundant between young and old adults.

## Discussion

Our findings are consistent with the hypothesis that changes in the gelada gut microbiome may help animals cope with seasonal changes in food availability and thermoregulatory demands. First, the gelada gut microbiome was highly plastic and responded rapidly to seasonal fluctuations in climate—particularly rainfall (a proxy for available foods). Second, an increase in predicted bacterial functions involved in energy, amino acid, and lipid metabolism during both drier and colder periods suggested increased production of SCFAs, and more efficient digestion during energetically and thermoregulatory challenging periods. We further found that individual identity and social group explained nearly a third of the variation of the gelada microbiome, while other individual traits such as sex, reproductive state, and age had little effect on gut microbiome composition and function.

Rainfall was the strongest ecological factor influencing changes in the gelada gut microbiome, explaining ~ 3.3% of overall microbiome composition. In particular, cellulolytic/fibrolytic and fermentative bacterial taxa increased during wetter periods when grass, which is mostly composed of cellulose, was the primary food source, while amylolytic and methanogenic bacterial taxa increased during drier periods, when geladas incorporated more starch (i.e., amylose) and lignified food into their diet. This effect of rainfall on the gut microbiome was strong, despite the fact that geladas exhibit only moderate dietary changes across season (i.e., from only grass to less grass and more underground organs—but from the same plant species) compared to other mammals living in more seasonal environments, e.g., that switch from ripe fruits to more folivorous diets [7, 52, 53]. This pattern highlights the importance of the gut microbiome for geladas in processing their unique diet across seasons.

The efficiency of grass digestion in wet periods seems to rely on a syntropy between the first cellulolytic degraders (*Ruminococcaceae*, *Lachnospiraceae*, *Fibrobacteraceae*, *Spirochaetes*) and a high diversity of secondary fermenters (*Prevotellaceae* and *Bacteroidales*), which all increase in abundance during the wet season. The first degraders attach to the plant cell walls and hydrolyze cellulose, hemicellulose, and xylan into smaller polysaccharides and oligosaccharides [12, 51], while secondary fermenters ferment those soluble polysaccharides into more simple sugars [10, 54]. *Ruminococcaceae* and *Lachnospiraceae* are the two main cellulolytic taxa in the mammalian gut and commonly increase in prevalence when animals eat more leaves and plants [7, 29]. In terms of secondary fermenters, *Prevotella* are widely known for their role in breaking down non-cellulosic polysaccharides and pectin [10, 12]. They are the major constituent (~ 70%) of rumen bacteria [55], and commonly increase in high fiber or fruit diets [29, 53, 56, 57]*.* Members of *Bacteroidales*—and particularly from the *Bacteroides* genus—have some of the largest repertoires of carbohydrate degrading activities and are able to ferment a broad range of plant polysaccharides [10, 58–60]. The increase in these cellulolytic/fibrolytic taxa and the high versatility of the secondary fermenters likely allow geladas to optimally extract nutrients from grasses eaten during wet periods.

In contrast, during drier periods, when geladas relied more on underground storage organs, we found a corresponding increase in microbial families involved in amylolytic and saccharolytic activities (*Succinivibrionaceae*, *Streptococcaceae*, *Christensenellaceae*). Interestingly, *Succinivibrionaceae* also increased during periods of energetic stress in Tibetan macaques (*Macaca thibetana*) [26] and during the dry season in the Hazda hunter gatherers of Tanzania [61], suggesting that it might help hosts cope with diet-related energy shortfalls. The gelada microbiome during the dry season was also characterized by an increase in *Methanobrevibacter*, a genus containing hydrogenotrophic archaea that converts hydrogen and formate into methane [62]. The simultaneous enrichment of efficient hydrogen-producers (e.g., *Christensenellaceae* [63], *Hydrogenoanaerobacterium*: [64]) and formate-producers (*Succinivibrionaceae*: [65]), combined with methanogens during the dry season suggest that these taxa work together in syntropy to improve the efficiency of polysaccharide fermentation from starch in the gut in dry periods [66, 67]. In mice and humans, a higher abundance of methanogenic archaea increases calorie harvest from diet, facilitates SCFA production by other fermentative bacteria, and stimulates lipogenesis [66–69].

Finally, drier periods were also characterized by a large increase in the *RFP12* family (i.e., ~ 30% versus ~ 18% in wetter periods) from the *Kiritimatiellaeota* phylum. The *RFP12* family remains poorly characterized but is increasingly recognized as being a keystone bacterial group in the hindgut of horses (*Equus ferus caballus*) [70–72], and a common inhabitant of the rumen of sheep (*Ovis aries*) or cattle [73–75]. The bacterial sequences belonging to the *RFP12* family found in the gelada feces were, on average, 97% similar to bacterial sequences found in the feces of various hindgut herbivorous fermenters [48] and, to a lesser extent, in the rumen of foregut fermenters (Table S3). This suggests that the *RFP12* family plays a particularly important, but thus far uncharacterized, digestive role for herbivorous species. In the case of geladas, it further suggests that it might be a keystone bacterial group for the digestion of some underground food components commonly eaten by the geladas during dry periods.

Overall, the gut microbes present in geladas are poorly represented by reference bacterial genomes in the two most common microbial databases: SILVA (updated 2017) [76] and Greengenes 13_8 (updated 2013) [77]. Taxonomic assignment of bacteria beyond the family level is relatively low (only 65% of our reads were assigned to genus level) compared to assignment of sequences found in captive animals and humans. This poor taxonomic characterization limits our ability to understand the metabolic activities of gut microbiome communities, and highlights the need to generate more molecular microbial data from a wider range of non-model and wild organisms. The *Kiritimatiellaeota* phylum (and in particular the *RFP12* family) would be a good candidate to focus on.

At the functional level, bacterial genes involved in energy, amino acid, and lipid metabolism increased in prevalence during the dry season. In particular, metabolic pathways linked to cellular respiration, methanogenesis, and carbon fixation pathways of prokaryotes became more common, strongly suggesting that both bacterial energy production and cellular activity were stimulated during this time. One interpretation of this data is that the increase in cellular activity simply reflects a dietary switch to starch, which is easier to hydrolyze than cellulose, and thus might more readily provoke a stimulation of bacterial activity and carbohydrate fermentation. Alternatively, the stimulation of bacterial energy metabolism and cellular activity could reflect a higher production of SCFAs by gut bacteria, supplying the host with additional energy in periods of nutrient restriction (when relying on fallback foods) [31, 78]. Similar increases in predicted bacterial energy metabolism have been found in energetically challenging environments (e.g., high altitude) in several other mammalian species and were correlated with higher SCFA production [31, 32]. Analysis of fecal SCFA profiles in geladas would help to identify if this is also the case in this high-altitude species. One caveat to our analysis is that the accuracy of the PICRUSt2 functional predictions may be lower than in other studies, especially when compared to published NSTI values in other mammals [50]. Previous studies of mammalian gut microbiomes conducted PICRUSt analyses on bacterial sequences grouped by OTUs, instead of ASVs. Because OTUs group sequences with 97% of similarity together, it removes 3% of genetic variation; and thus by definition OTUs will be closer to samples from reference genomes, potentially leading to lower NSTI values. Thus, although our analyses are reflecting only a limited portion of the bacterial community of geladas, it is not necessarily a worse representation than found in previous studies clustering bacteria by OTUs. Overall, while our predicted functions might give us a broad profile of the differences in metabolic activities of gut bacteria in the dry and wet seasons, they are not as precise as functional characterization from metagenomic or metabolomic data.

While it is clear that the gelada diet shifts during drier periods, it remains unknown if (and to what extent) geladas are nutritionally or energetically constrained during this time. Grass availability declines and geladas spend more time foraging and digging for underground plant parts during the dry season [35, 42]. Such underground foods are usually considered fallback foods because individuals rely on them only when grass is less available and because they require long processing times [35, 38]. However, one study [42] found that geladas obtain just as much, or even more, calories from underground storage organs as they do from grass. Whether this increased caloric intake is offset by increased foraging costs is currently unknown. However, even if increased foraging costs were demonstrated, our data suggests that the gut microbiota may increase digestive efficiency from starchy food and thereby help geladas maintain or improve energetic status during the dry season. Future studies on seasonal changes in energy balance will help resolve this issue.

In contrast to the effect of rainfall, we found mixed evidence for the effect of temperature on the gut microbiome. Temperature only explained ~ 0.33% of variation in the gelada gut microbiome composition. Furthermore, few taxa shifted in abundance between the coldest and hottest months, and most taxa affected by temperature were also affected by rainfall. This might be explained by the fact that rainfall (and thus diet) covaries with temperature to some extent (Pearson’s correlation coefficient = 0.20): geladas rely the most on underground foods in the hot-dry season (Feb. to May) and the most on grass on the cold-wet season (Jun. to Sep.) [35]. The cold-dry season (Oct. to Jan.), however, displays a mixed pattern of diet and temperature: grass availability is still high in Oct.-Nov. (following the rainy season) but decreases markedly in Dec.-Jan. [35, 41]. These 2 months are thus characterized by the introduction of underground foods in the diet and are also the coldest months of the year, making them likely the most challenging times for geladas (compounding nutritional and thermoregulatory challenges). Accordingly, cold periods were characterized by an increase in two amylolytic and lactate-producing taxa (*Streptococcus*, *Lactobacillus*), presumably to more efficiently extract starch from the underground foods. At the functional level, the energy and lipid metabolism of bacteria were also stimulated in the cold months, further suggesting some role of gut bacteria in stimulating host digestive efficiency and energy metabolism during thermoregulatory-demanding times.

These seasonal changes that increase energy production during colder periods may come at some cost. Such trade-offs have been proposed where shifts that benefit one aspect of host physiology consequently lead to a decrease in other microbes that may also be necessary for the host. For example, microbes that promote host digestive efficiency and energy metabolism may also promote inflammation or even suppress immune function [33, 79]. We did not detect any obvious evidence of these tradeoffs in geladas, but future work that incorporates detailed host immunological and functional microbial data is needed to help determine if such trade-offs exist.

Finally, the present study found that the gelada gut microbiome was largely explained by individual identity (20%), a pattern consistent with data from a range of vertebrates [40, 80–82], including humans [83, 84]. However, the effect of social group was lower in geladas than reported for other social mammals (geladas: 6.0% vs., e.g., yellow baboon, *Papio cynocephalus*: 18.6% of variation explained [85], black howler monkey: 14% [86], ring-tailed lemurs, *Lemur catta*: 21% [87], Welsh Mountain ponies: 14%: [82]). The combination of large individual effects with weak social group effects closely resembles data reported for the Guassa gelada population [40], suggesting a general, but consistent pattern. The weak unit-level effects may result from the unique social system of geladas: Because social units often aggregate into large bands whose composition changes regularly, geladas may have a higher rate of inter-unit microbial transmission compared with other primates. Future studies should explore in more detail the intra-individual fluctuation in gut microbiome composition, and whether group differences in ranging patterns may explain these differences.

Other individual predictors, namely, age, sex, and female reproductive state, had a very limited effect on the gut microbiome, mirroring results in other mammals (yellow baboons: [28, 85], ring-tailed lemurs: [87], Verreaux’s sifakas, *Propithecus verreauxi*: [29], chimpanzees, *Pan troglodytes schweinfurthii*: [88], rhesus monkeys, *Macaca mulatta*: [89], Welsh Mountain ponies [82], domestic dog, *Canis lupus familiaris*: [90], but see black howler monkeys: [34] or Egyptian fruit bats, *Rousettus aegyptiacus*: [80]). Although female geladas harbored higher microbial richness than males, this resulted in minimal differences in gut microbial composition and predicted function. Compared to males, females had higher abundance of *Proteobacteria* and *Lactobacillus*. These two bacterial taxa were previously reported to increase during pregnancy and lactation in humans and non-human primates [27, 91], and act as early colonizers of the infant gut [92–94]. Additionally, pregnant female geladas harbored more *Helicobacter*, a potentially pathogenic genus [95, 96]. An increase in potentially pathogenic microbes in pregnant females was also observed in black howler monkeys [34] and was hypothesized to be the consequence of a trade-off between reproduction and immunity. These dynamics warrant further investigation.

Overall, the gut microbiome of geladas seems to be highly plastic and can respond rapidly to changes in host diet and thermoregulatory demands. Stimulation of bacteria cellular activity could allow geladas to maintain adequate or even improved energetic balance during dry and cold periods. Our study adds to an increasing body of literature suggesting that the gut microbiota is an important system providing dietary and metabolic flexibility for the host and might be a key factor influencing the acclimatization to changing environments [8, 48, 97, 98]. In addition to fostering phenotypic plasticity, the gut microbiome is increasingly hypothesized to contribute to host evolution and speciation [97–99] given the strong host phylogenetic signal in mammalian microbiome composition and function [100, 101] and evidence of microbiome heritability [102–104]. To the extent that microbiomes affect host phenotypes under selection, they will also affect host evolutionary trajectories. In the case of geladas, a shift in gut microbiome composition was probably an important adaptive mechanism that allowed members of the *Theropithecus* genus to adopt a specialized dietary niche and diversify rapidly from *Papio* ~ 5 million years ago [105]. Contrary to host adaptive genetic mutations, which occur over the course of many generations, the gut microbiota can shift in response to changes in host diet in a matter of days [9]. Given that the common ancestor of *Theropithecus* and *Papio* was omnivorous [106, 107], dietary flexibility provided by the gut microbiome may have been an important first step allowing members of *Theropithecus* to exploit new grassland habitats in East Africa, leading to the evolution of a specialized diet and, ultimately, further genetic and phenotypic adaptation.

## Conclusion

The gut microbiome of geladas is plastic and responds rapidly to changes in host diet and thermoregulatory demands. These shifts appear to help geladas cope with seasonal fluctuations in nutrient intake and energy homeostasis, potentially allowing them to maintain energy balance in challenging seasonal environments. Our study adds to an increasing body of literature suggesting that the gut microbiota is an important system that provides metabolic flexibility for the host, and is key to helping hosts adapt and shift to fluctuating environments. Future research in geladas and other animals will further uncover how the gut microbiota allows hosts to occupy unique dietary niches, potentially allowing host lineages to expand into new habitats, and facilitating speciation.

## Material and methods

### Study population and fecal sample collection

We collected fecal samples from a wild population of geladas living in the Simien Mountains National Park, in northern Ethiopia (13° 15′ N, 38° 00′ E). Samples were collected over a 4-year period between Jan. 2015 and Feb. 2019. Geladas live in multi-level societies, where reproductive units (comprising a leader male, several adult females, their offspring, and occasionally 1–2 follower males) and bachelor groups (comprising between 1 and 10 adult males) form the smallest levels of the society that forage and sleep together in a “band” sharing the same home range [108]. Since Jan. 2006, the Simien Mountains Gelada Research Project (SMGRP) has collected demographic and behavioral data on over 200 individuals from two bands. All individuals are habituated to human observers on foot and are individually recognizable. Dates of birth of individuals were established using a combination of known (*N* = 42) and estimated (*N* = 89) birth dates. Estimated birth dates were calculated by using the mean individual age at major life-history milestones in our population (e.g., sexual maturation or first birth for females and canine eruption for males) [109, 110]. Birth dates of unknown immigrant males were estimated using an established protocol based on body size and other age-related morphological characteristics [110]. Here, we focused only on samples from adult males and females. Adult males were included when they reached 7 years of age. At this age, males have reached adult body size in stature but not in weight [110, 111], and most males have dispersed into a non-natal group (i.e., 96% of our male samples, males could thus be leaders, followers, bachelors, or natals). Adult females were included after they had experienced their first sex skin swelling, a marker of reproductive maturation (which is around 4.65 years old in our population [109]).

Fecal samples of known adult and subadult male and female subjects were collected regularly and opportunistically during the study period. Immediately upon defecation, approximately 1.5 g of feces was collected in 3 ml of RNA later [112, 113], stored at room temperature for up to 2 months, and subsequently shipped to the University of Washington (UW). At UW, samples were stored at −80 °C until the sequencing libraries were prepared. A total of 758 samples (620 female samples, 138 male samples) were collected from 131 individuals (83 females, 48 males) (mean ± SD = 5.79 ± 6.14 samples per individual, range = 1-21) from 28 reproductive units and 4 bachelors groups (mean ± SD = 4.69 ± 2.97 number of individuals sampled per unit, range = 1-11).

The reproductive state of females at the date of sample collection was assigned based on daily monitoring of individuals for the status of sex skin swellings and the birth of infants. We assigned the three reproductive states as follows: (1) Cycling began at the first sign of postpartum sex skin swelling and ended when a female conceived—with conception defined as 183 days (mean gestation length) before the birth of a subsequent infant [109]. (2) Pregnancy started on the date of conception and ended the day before parturition. (3) Finally, lactation started on the day of parturition and ended the day before the female’s first postpartum swelling. Lactating females were further categorized as being in early lactation (infant < 1 year old) or late lactation (infant > 1 year old). When testing the effect of reproductive state, late lactating females were removed from the lactating category to include only females that were still nursing at the time of sample collection (females resume cycling when infants are ~ 1.5 years old in our population, which is presumably accompanied by infant weaning around the same time [109]). Furthermore, because pregnant females can abort their fetus during male takeover of their reproductive unit [114], some pregnancies might have been misidentified as cycling based on our method of back-calculating from the date of birth. We therefore removed cycling females that experienced a takeover in the previous 6 months before the date of sample collection (*N* = 55 samples) to avoid any misclassification of reproductive state in our analyses.

### Study site and climatic data

The study area is located at 3200 m above sea level and is characterized as an Afroalpine grassland ecosystem, consisting of grassland plateaus, scrublands, and Ericaceous forests [115]. Fecal samples were collected across the year, with roughly equal coverage across seasons (244 in cold-dry, 298 in cold-wet, and 216 in hot-dry season as defined above). As part of the long-term monitoring of the SMGRP, daily cumulative rainfall and minimum and maximum temperature are recorded on a near-daily basis. We used the total cumulative rainfall over the 30 days prior to the date of fecal sample collection as a proxy for grass availability at the time of sample collection [35]. In addition, we used the average minimum daily temperatures in the 30 days preceding the date of sample collection as a proxy of thermoregulatory constraints. The average minimum temperature is less correlated with cumulative monthly rainfall than the average maximum temperature in the previous 30 days (correlation coefficient: 0.25 versus −0.56) and, more importantly, is more likely to reflect the physiological effect of thermoregulation on the body [41, 44].

### DNA extraction, sequencing, and data processing

We prepared 16S sequencing libraries using the protocols developed and optimized by the Earth Microbiome Project and the University of Minnesota Genomics Core (UMGC [116];). We extracted microbial DNA from the fecal samples using Qiagen’s PowerLyzer PowerSoil DNA Isolation kit (Qiagen #12855) following the standard protocol. We amplified the hypervariable V4 region of the 16S rRNA gene using PCR primer set 515F (TCGTCGGCAGCGTCAGATGTGTATAAGAGACAGGTGYCAGCMGCCGCGGTAA) and 806R (GTCTCGTGGGCTCGGAGATGTGTATAAGAGACAGGGACTACNVGGGTWTCTAA

T) from The Human Microbiome Project and a dual**-**indexing approach [116]. Details of the amplification protocol can be accessed at https://smack-lab.com/protocols/. The first PCR round aimed at amplifying the V4 region. Each 25 μl PCR reaction well consisted of 12.5 μl of Nebnext Ultra II Q5 mastermix, 1.0 μl of each primer, and 25 ng of total DNA in 10.5 μl of nuclease-free water. PCR was performed in an Eppendorf thermocycler with a 100 °C heated lid using the following cycling steps: an initial denaturing for 5 min at 95 °C; followed by 15 cycles of 20 s at 98 °C, 15 s at 62 °C, 60 s at 72 °C, and a final hold at 4 °C. We cleaned up the PCR reaction with a 2:1 ratio of SPRI beads to PCR amplified DNA. The second PCR round aimed at adding a unique index primer combination to molecularly barcode each sample. We took 4 μl of product from the first PCR and added 6 μl of Nebnext Ultra II Q5 mastermix and 1 μl of n5 and n7 indexing primers, with each sample being assigned a unique n5/n7 index primer combination. This 12 μl reaction was placed in an Eppendorf thermocycler with a 100 °C heated lid, denatured for 5 min at 95 °C, and amplified with 10 cycles of 20 s at 98 °C, 15 s at 55 °C, and 60 s at 72 °C with a final hold at 4 °C. After a 2:1 SPRI bead clean-up, amplification of the V4 region was confirmed in a few samples using an AATI fragment analyzer, and all libraries were quantified using a qubit fluorometer. The libraries were then pooled in roughly equimolar amounts (each with their own unique indexing primer combination), spiked with 10% PhiX to increase library complexity, and sequenced together on a single Illumina NovaSeq 6000 SP 250 bp paired-end sequence flowcell.

We analyzed the resulting data using the Quantitative Insights Into Microbial Ecology 2 (QIIME2) platform [117, 118]. After trimming low-quality bases from the de-multiplexed reads, we merged overlapping paired-end reads, and denoised the sequencing data by filtering and correcting Illumina amplicon sequencing errors using the Divisive Amplicon Denoising Algorithm 2 (DADA2: [119]) plugin incorporated in QIIME2. DADA2 infers sequences exactly resulting in amplicon sequence variants (ASVs). Forward and reverse reads were trimmed to 220 and 180 bases, respectively, to remove the low**-**quality portion of the sequences. The forward and reverse reads were then merged together and chimeric sequences were removed. Only samples with more than 20,000 reads were retained for analyses (following observation of rarefaction curves, Figure S12). After filtering, trimming, merging, and chimera removal, we retained a total of 348,390,395 reads across the 758 fecal samples (459,618 ± 815,020 reads per sample, range = 20,109-10,735,588). ASVs were taxonomically assigned using the q2**-**feature classifier in QIIME2 against version 132 of the SILVA database (updated December 2017) [76] based on 100% similarity. Uninformative taxonomic assignments of ASVs found in SILVA (e.g., “wallaby metagenome,” “unassigned bacteria,” etc.) were converted to “NA” to simplify analyses at higher taxonomic levels. All ASVs belonging to the order *WCHB1-41* (phylum *Kiritimatiellaeota*) were not assigned at the family level in the SILVA classification. However, in the Greengene classification (version 13_8) [77], all ASVs from this order in the gelada gut were assigned to the *RFP12* family. Thus, we attribute the family *RFP12* to all ASVs from the order *WCHB1-41* in SILVA classification. The 78 ASVs from the *RFP12* family were found in particular high proportion in the gelada feces, but we did not have taxonomic information about those bacterial sequences below the family level. We thus mined previously published data to identify bacterial sequences most similar to the 78 *RPF12* ASVs in geladas by using the NCBI BLAST search engine [46, 47]. For each of those 78 ASV, we searched for the most similar nucleotide sequences previously published, extracted its top five hits (i.e., most similar sequences based on the *E* value), and summarized the distribution of those top hits per ASV across the 78 ASVs (including in which animal species those hits were found, see Table S3). The five top hits had an average of 97% nucleotide similarity (range, 93-100%) with their respective similar gelada ASVs.

### Statistical analyses

The count and taxonomy files generated by QIIME2 were imported into R version 3.5.2 [120] using the qiime2R package [121] and analyzed using the phyloseq package [122]. The majority of the 19,606 ASVs in our dataset were found at very low frequency or only in one sample (71% of ASVs were found in only one sample and 6.2% of ASVs were not assigned at the phylum level). Thus, we further filtered the count table to retain only ASVs that had at least 500 reads in total in the dataset (i.e., 0.00014% relative abundance) to eliminate potentially artifactual sequences. With this filtering criteria, only 3295 ASVs remained, with all of them assigned at the phylum level and most (97%) observed in at least two samples (Figure S13). The use of rarefaction (i.e., subsampling of the read count in each sample to a common sequencing depth) has been discouraged due to the loss of information and precision [123], as well as the use of count normalization methods from the RNA-seq field (e.g., DESeq2 or edgeR). However, microbiome datasets are more sparse (zero-inflated) and more asymmetrical than genetic expression datasets [124, 125]. Thus, we used a compositional approach when possible (e.g., centered-log-ratio normalization of the counts and Aitchison distance for beta diversity analysis) [125, 126], controlling for sample sequencing depth in multivariate analyses to account for repeated samples from the same individual.

We replicated alpha- and beta-diversity analyses using traditional rarefaction methods to facilitate comparisons with other studies. To generate the rarefied dataset, we randomly sampled 20,000 reads from the raw fastq files of each sample and processed this new rarefied dataset into the DADA2 pipeline. This dataset was further filtered to remove the low-frequency ASVs (i.e., ASVs not included in the pool of 3295 ASVs retained in the full dataset). This resulted in a dataset containing the same 758 samples, with 2853 ASVs and with relatively homogenous sequencing depth (18205 ± 1415 reads per sample, range = 7460-19444). We rarefied reads from the raw fastq files prior to DADA2 processing instead of the traditional approach of rarefying after DADA2 using the rarefy_even_depth function of the phyloseq package [122] because the traditional approach did not eliminate the effect of sequencing depth (post-DADA2) on the alpha diversity metrics in the rarefied dataset. Subsampling the raw fastq files allowed us to better generate indices of alpha diversity per sample that did not depend on the total number of reads obtained initially for the sample.

All mixed models described below were run using either the lmer (for linear mixed models, LMMs) or glmer (for binomial and negative binomial generalized linear mixed models, GLMMs) functions of the lme4 package [127]. All quantitative variables (i.e., cumulative rainfall, averaged temperature, and age) were z-transformed to have a mean of zero and a standard deviation of one to facilitate model convergence. The significance of the fixed factors was tested using a likelihood ratio test, LRT (assuming an asymptotic chi-square distribution of the test statistic) via the drop1 function. To test for significant pairwise differences between levels of multilevel categorical variables (i.e., reproductive state), post hoc Tukey’s Honest Significant Difference tests were carried out using the multcomp package in R [128].

#### Alpha-diversity analyses

We calculated three measures of alpha diversity: observed richness (the total number of different ASVs in a sample), Shannon diversity index (accounts for both richness and evenness of ASVs in a sample), and Faith’s phylogenetic diversity (accounts for phylogenetic distance between bacterial species, using the picante package [129]). We modeled each alpha diversity metric using linear mixed models: (i) as a function of age, sex, cumulative monthly rainfall, average monthly minimum temperature, and sequencing depth of the sample (*N* = 758 samples), and (ii) as a function of reproductive state (cycling, early lactating, and pregnant), age, cumulative monthly rainfall, and average monthly minimum temperature in samples collected from females (*N* = 439). Individual identity and unit membership were included as random effects to control for individual and unit repetition across samples. We also ran the same models on the rarefied dataset (Table S16).

#### Beta-diversity analyses

We then assessed how the same predictors were associated with between-sample community dissimilarity. To account for differences in sequencing depth between samples, the counts were normalized using the centered-log-ratio (CLR) method (and using a pseudo count of 0.65 for zero counts) from the “compositions” package [130]. We then calculated the Aitchison distance between samples (i.e., simply the Euclidean distance between samples after clr transformation of the counts) [131] and conducted a Principal Component Analysis (PCA) (function “prcomp”) to visually represent between-samples dissimilarity according to the predictors. This approach has been recommended for microbiome datasets [125] and allows for the projection of each sample onto individual principal components (PCS) and the variable loadings of ASVs onto each PC. While the first axis of variation correlated mostly with rainfall (Fig. 2b), the second PCA axis was correlated with sequencing depth and explained 11% of the variation (Figure S14). We used Permutational Multivariate Analysis of Variance (PERMANOVA) tests to assess the effect of the predictors on the Aitchison distance between samples (using 10,000 permutations and the “adonis2” function from the “vegan” package [132]). We ran three different models: (1) including all samples where we tested only the effect of individual identity and sequencing depth, (2) including all samples where we tested the effect of unit, age, sex, cumulative monthly rainfall, average monthly minimum temperature, and sequencing depth of the sample, and (3) including only female samples where we tested the effect of unit, reproductive state, age, cumulative monthly rainfall, and average monthly minimum temperature. In models 2 and 3, individual identity was included as a blocking factor (“strata”) to control for repeated sampling. We also replicated beta diversity analysis on the rarefied dataset. We ran PERMANOVA tests using three complementary pairwise dissimilarity metrics (Bray-Curtis distance, unweighted and weighted UniFrac distances) to assess between-sample variation according to the same predictors (the same three models). Beta diversity results remained qualitatively similar (Table S17).

#### Differential abundance testing

We examined how our predictors were associated with differential abundance of bacteria (at the phylum, class, order, family, and genus levels) using negative binomial GLMMs. Compared to LMMs, negative binomial mixed models are better equipped to handle over-dispersed and zero-inflated distributions that often characterize microbiome datasets [133]. They also facilitate tests of several independent predictors while taking into account longitudinal designs including random effects. We first aggregated the counts (i.e., the number of reads per taxa and per sample) at the taxonomic level of interest. Only taxa that had an average relative abundance across samples ≥ 0.01% were tested. Then, for a given taxa, the count per sample was modeled as a function of (1) age, sex, cumulative monthly rainfall and averaged monthly minimum temperature (all samples), or (2) female reproductive state, age, cumulative monthly rainfall, and averaged monthly minimum temperature (female samples only). The log-transformed number of reads per sample was included as an offset term to control for variation in sequencing depth across samples. Individual identity and unit membership were included as random effects in all models. When negative binomial models failed to converge in some taxa, we converted the counts in presence/absence and modeled them with binomial GLMMs. Benjamini-Hochberg corrected p-values < 0.05 were considered statistically significant.

#### Functional profiling of microbiota

We estimated the bacterial and archaeal genes present in the metagenomes of each sample using Phylogenetic Investigation of Communities by Reconstruction of Unobserved States version 2 (PICRUSt2) [50]. In brief, ASVs were aligned to reference sequences using HMMER [134] and placed into a reference tree using EPA-NG [135] and Gappa [136]. PICRUSt2 normalizes for multiple 16S gene copies in bacteria using castor, a hidden state prediction tool [137]. The normalized data were used to predict gene family profiles, and mapped onto gene pathways using MinPath [138]. We followed the default protocols outlined on the PICRUSt2 GitHub page (https://github.com/picrust/picrust2/wiki). We investigated the predicted gene families using the Kyoto Encyclopedia of Genes and Genomes (KEGG) Orthology (KO) database. The accuracy of the PICRUSt2 predictions for each sample was assessed by calculating the weighted Nearest Sequence Taxon Index (NSTI) score, a measure of how similar the bacteria from the sample are to reference genome sequences. Five ASVs (out of 3295) had a NSTI score > 2 and were removed from our final predictions. The association between the relative abundance of functional categories as estimated by PICRUSt2 and the predictors (on all samples or female samples only) were examined using LMMs. Only functional pathways that had ≥ 0.1% relative abundance across samples were tested. Individual identity and unit membership were included as random effects in all models.

## Supplementary Information

**Additional file 1: Table S1.** Taxonomic composition of the gelada gut at the phylum, class, order, family and genus levels. The mean relative abundance and prevalence (% of the samples with each taxon) of bacterial taxa are indicated. **Table S2.** Taxonomic composition of the core ASVs (i.e. present in at least 90% of samples) in the gelada gut at the order level. **Table S3.** Summary of the most similar bacterial sequences to the 78 ASVs belonging to the *RFP12* family found in geladas feces. For each gelada *RFP12* ASV, we searched the most similar nucleotide sequences found previously on NCBI BLAST, extracted its top five hits (i.e. most similar sequences based on Evalue) and summarized the distribution of those top hits across the 78 ASVs (identifying those animals in which similar sequences were found). **Table S4.** Predictors of observed richness and Faith’s phylogenetic diversity (PD). Estimates with p-values <0.05 are highlighted in bold. **Table S5.** Loading scores of ASVs on the first principal component**.** Positive loadings correspond to wetter periods, while negative loadings correspond to drier periods. **Table S6.** Taxonomic distribution of seasonally differentially abundant taxa. Only taxa with a PC loading scores > 0.4 and <-0.4 are included. **Table S7.** Differential abundance results for all taxa. Results were obtained by fitting negative binomial GLMMs for each taxa, controlling for individual identity and unit membership. The estimate and p-values of fixed effects are reported. Due to some negative binomial models that did not converge, taxa preceded by a “*” were modeled with a binomial GLMM, with presence/absence as the outcome variable. **Table S8.** Differential abundance results for KEGG pathways level 2. Model results were obtained by fitting LMMs on each pathway, while controlling for individual identity and unit membership. The estimate and p-value of the fixed effects are reported. **Table S9.** Differential abundance results for KEGG pathways level 3. Model results were obtained by fitting LMMs on each pathway, controlling for individual identity and unit membership. The estimate and p-value of the fixed effects are reported. **Table S10.** Predictors of Shannon index, observed richness and Faith’s phylogenetic diversity (PD) in females only. Estimates with p-values <0.05 are highlighted in bold. **Table S11.** Predictors of the structure of the female gelada gut microbiome. We carried out a PERMANOVA using 10,000 permutations and the Aitchison dissimilarity distance between samples. **Table S12**. Number of differentially abundant taxa for each predictor in the female samples. **Table S13**. Differential abundance results for female samples at five taxonomic levels. **Table S14.** Differential abundance results for KEGG pathways level 2 for female samples. Model results were obtained by fitting LMMs on each pathway, while controlling for individual identity and unit membership. The estimate and p-value of the fixed effects are reported. **Table S15.** Differential abundance results for KEGG pathways level 2 for female samples. Model results were obtained by fitting LMMs on each pathway, while controlling for individual identity and unit membership. The estimate and p-value of the fixed effects are reported. **Table S16.** Predictors of Shannon index, observed richness, and Faith’s phylogenetic diversity (PD) on (1) all samples and (2) female samples on a rarefied dataset. Parameters and tests are based on 758/439 samples and 131/70 individuals in all models. The LMMs were performed controlling for individual identity and unit membership. The 95% confidence intervals that do not cross zero and p-values of statistically significant results are highlighted in bold. **Table S17.** Results of PERMANOVA testing for the effects that significantly structure the gut microbiome of geladas on a rarefied dataset for (1) all samples or (2) female samples only. We used Bray Curtis, unweighted Unifrac or weighted Unifrac distances to assess between-sample dissimilarity. 10000 permutations were carried out and individual identity was added as a strata in the model. The R-squared values indicate the amount of between-sample variation explained by each variable.

**Additional file 2: Supplemental Figure 1.** Taxonomic composition of the gelada gut microbiome at the phylum and family levels. Relative abundance (A) of all bacterial phyla and (B) of the 24 most abundant families (mean relative abundance>0.02%) in the gelada feces. The y-axis is on a log10 scale to better represent the variation across samples. The median and median absolute deviation (error limit) are represented in orange. **Supplemental Figure 2.** Genus composition of the gelada gut. Relative abundance of the 38 most abundant genera (mean relative abundance>0.01%) in the gelada feces. The tick marks on the y-axis are spaced on a log10 scale. The median and median absolute deviation (error limit) are represented in orange. **Supplemental Figure 3.** Rainfall is not associated with Observed richness and Faith’s phylogenetic diversity. Partial residual plot of (A) Observed richness and (B) Faith’s phylogenetic diversity (PD) according to cumulative rainfall (in mm). Black dots represent the partial residuals from the LMM (i.e. showing the association between cumulative rainfall and alpha diversity, while controlling for all other predictors). The blue line and confidence intervals come from a linear regression (for representation only). One and 5 outlier samples (with a particularly low diversity) were omitted for panel A and B respectively for clarity of representation. **Supplemental Figure 4.** Bacterial functional pathways that significantly associated with cumulative rainfall at KO level 3. The estimate of the cumulative rainfall effect comes from a LMM fitted on the relative abundance of each pathway per sample. Only pathways with p_BH_ < 0.05 were considered significant. For ease of representation, only pathways with effect sizes > |0.006| are represented. The full list can be found in Table S9. Classification of KO level 3 pathways in broader categories were based on their KO level 2 assignment, with a few changes made for clarity of representation. Level 3 pathways from *Metabolism of Other Amino Acids* (level 2) were reclassified in the “Amino Acid Metabolism”, *Translation proteins* and *Replication, recombination and repair proteins* (both level 3 and initially in Genetic Information Processing at level 2) were reclassified in “Transcription & Translation" and "Replication and Repair" respectively. The category “Membrane Transport & Cellular Signalling" regroups pathways from “Membrane Transport” and the other pathways from “Cellular Processes and Signaling”. **Supplemental Figure 5.** Rainfall predicts the functional profile of the gut microbiome. Relative abundance of ten functional pathways (at KO level 3) that are enhanced (A) during the wet season and (B) during the dry season. Note that the tick marks on the y-axis are spaced on a log10 scale. The blue line and confidence intervals come from a linear regression (for representation only). The significance of the rainfall effect effects per pathway have been estimated using LMMs including individual and unit membership as random effects. **Supplemental Figure 6**. Small effect of ambient temperature on the gelada gut microbiome. (A) Partial residual plots of the three alpha diversity indices (Shannon index, Observed richness and Faith’s phylogenetic diversity) according to the average minimum temperature in the previous month of sample collection (in ^o^C). Black dots represent the partial residuals from the LMM (i.e. showing the association between temperature and alpha diversity, while controlling for all other predictors). The blue line and confidence intervals come from a linear regression (for representation only). For clarity of representation, 9, 1, and 5 outlier samples (with a particularly low diversity) were omitted for Shannon, richness and Faith’s PD, respectively. (B) Visualization of between-sample dissimilarity (based on Aitchison distance) on the first and second principal component according to minimum temperature. (C) Compositional barplot of the five most abundant phyla in the cold (i.e. <8^o^C in the past month, N=191) and hot (>8^o^C in the past month, N=567) samples (minimum temperature was converted to a categorical variable for representation purposes). **Supplemental Figure 7**. Genera that significantly associated with average minimum temperature. The estimate for the effect of temperature for each taxa comes from a negative binomial GLMM controlling for sample sequencing depth as an offset factor, and including individual and unit membership as random effects. Only taxa with p_BH_ < 0.05 were considered significant. The full list can be found in Table S7. **Supplemental Figure 8**. Bacterial pathways that are differentially abundant according to average minimum temperature at KO (A) level 2 and (B) level 3. The estimate comes from a LMM fitted on the relative abundance of each pathway per sample. Only pathways with p_BH_ < 0.05 were considered significant. For ease of representation on panel B, only pathways with effect size > |0.002| were represented. The full list can be found in Table S8. **Supplemental Figure 9**. Effect of sex on the gelada gut microbiome. (A) Partial residual plots of the three alpha diversity indices (Shannon index, Observed richness and Faith’s phylogenetic diversity) according to the sex of the sampled individual. Black dots represent partial residuals of the LMM. The median and median absolute deviation (error limit) of the distribution are represented in orange. Ten, 1 and 5 outlier samples (with a particularly low diversity) were omitted for Shannon, richness and Faith’s PD respectively for clarity of representation. (B) Visualization of between-sample dissimilarity (based on Aitchison distance) on the first and second principal component according to sex. (C) Compositional barplot of the five most abundant phyla in male (N=138) and female (N=620) samples. **Supplemental Figure 10**. Effect of female reproductive state on the gelada gut microbiome. (A) Partial residual plots of the three alpha diversity indices (Shannon index, Observed richness and Faith’s phylogenetic diversity) according to the reproductive state of the sampled female. Black dots represent partial residuals of the LMM. The median and median absolute deviation (error limit) of the distribution are represented in orange. Ten, 1 and 5 outlier samples (with a particularly low diversity) were omitted respectively for clarity of representation. (B) Visualization of between-sample dissimilarity (based on Aitchison distance) on the first and second principal component according to reproductive state. (C) Compositional barplot of the five most abundant phyla in pregnant (N=61), lactating (N=346) and cycling (N=158) female samples. **Supplemental Figure 11.** Effect of age on the gelada gut microbiome. (A) Partial residual plots of the three alpha diversity indices (Shannon index, Observed richness and Faith’s phylogenetic diversity) according to the age of individuals at the date of sample collection (in years). Black dots represent the partial residuals from the GLMM (i.e. showing the association between age and alpha diversity, while controlling for all other predictors). The blue line and confidence intervals come from a linear regression (for representation only). Nine, 1 and 5 outlier samples (with a particularly low diversity) were omitted respectively for clarity of representation. (B) Visualization of between-sample dissimilarity (based on Aitchison distance) on the first and second principal component according to age. (C) Compositional barplot of the five most abundant phyla between young (<10 years old, N=215 samples), middle-aged (10 to 17 years old, N=420 samples) and old (>17 years old, N=123 samples) individuals (age was converted to a categorical variable for representation purposes only). **Supplemental Figure 12.** Rarefaction curves of samples. Only samples that had at least 20000 reads were included in this study. **Supplemental Figure 13.** 16S sequencing and dataset characteristics. (A) Distribution of the total number of reads per sample (the tick marks on the x-axis are spaced on a log10 scale). (B) Distribution of the total number of ASVs per sample. **Supplemental Figure 14.** Visualization of differences in the gut microbiome composition according to sequencing depth of the samples based on Aitchison distance dissimilarity matrix. Points represent individual samples.

## Data Availability

All 16S sequence data used in this study are available at the NCBI Sequence Read Archive (https://www.ncbi.nlm.nih.gov/) under BioProject ID PRJNA639843. Data and code (including how to run the QIIME2 pipeline on our data) are available at 10.5281/zenodo.3932310.
